# **Association of nutritional status indices with gastrointestinal symptoms in systemic sclerosis: a cross-sectional study**

**DOI:** 10.1007/s00296-024-05783-2

**Published:** 2025-01-08

**Authors:** Nuran Öz, Halise Hande Gezer, Yusuf Karabulut, Mehmet Tuncay Duruöz

**Affiliations:** 1 Physical Medicine and Rehabilitation Department, Rheumatology Division, Marmara University School of Medicine, Istanbul, Türkiye; 2 Internal Medicine Department, Rheumatology Division, Yıldırım Doruk Hospital, Bursa, Türkiye

**Keywords:** Systemic sclerosis, Prognostic nutritional index, The UCLA Scleroderma Clinical Research Consortium gastrointestinal tract 2.0, The Control of Nutritional Status (CONUT) score

## Abstract

Gastrointestinal (GI) involvement is highly prevalent in systemic sclerosis (SSc) and significantly affects patient quality of life and clinical outcomes. This study investigates the potential of undernutrition scores, namely the Control of Nutritional Status (CONUT) score and the Prognostic Nutrition Index (PNI), in predicting GI involvement in patients with SSc. A total of 82 patients diagnosed with SSc were enrolled in this cross-sectional study. Participants were evaluated using the UCLA Scleroderma Clinical Research Consortium Gastrointestinal Tract 2.0 (UCLA GIT 2.0) tool, which assesses the severity of GI symptoms and their impact on health-related quality of life. Malnutrition was assessed using CONUT and PNI scores derived from routine laboratory parameters. The correlation between these malnutrition indices and the UCLA GIT 2.0 scores was analyzed to determine the predictive value of malnutrition in GI involvement. The study found that patients with higher CONUT scores, indicating malnutrition, had significantly higher total UCLA GIT 2.0 scores. A moderate positive correlation was observed between CONUT scores and total UCLA GIT 2.0 scores (*r* =.539; *p* <.01), while a negative correlation was found between CONUT scores and PNI (*r* = −.513; *p* <.01). These findings suggest that malnutrition, as measured by CONUT and PNI, is associated with greater GI involvement in SSc. This study shows that malnutrition indices such as CONUT and PNI in SSc patients, together with the UCLA GIT 2.0 score, may serve as usefull predictors of GI involvment in routine clinical practice.

**Supplementary Information:**

The online version contains supplementary material available at 10.1007/s00296-024-05783-2.

## Key-points

(1) We investigated the relationship between the UCLA GIT 2.0 score, which assesses GI involvement in patients with systemic sclerosis (SSc), and the CONUT score and PNI, indicators of malnutrition.

(2) In the study, a negative correlation was detected between UCLA GIT 2.0 scores and CONUT score and a positive correlation was found with PNI.

(3) CONUT and PNI scores derived from routine laboratory parameters provide a simple and effective method to assess malnutrition and predict GI complications in SSc patients.

**Supplementary Information:**

The online version contains supplementary material available at 10.1007/s00296-024-05783-2.

## Introduction

Systemic Sclerosis (SSc) is a multisystem autoimmune disease characterised by vasculopathy, chronic inflammation, immune dysregulation and the subsequent development of fibrosis of the internal organs and skin [1]. Gastrointestinal (GI) involvement affects up to 90% of the population of patients with SSc, making it the most frequent site of visceral manifestation in the condition. The pathogenesis of GI symptoms in SSc is thought to stem from fundamental mechanisms including autoimmunity, inflammation, vasculopathy and fibrosis. Additionally, factors including medications, GI microbiota, dietary habits and concomitant health conditions are potentially contributing to these symptoms [2].

Various patient-reported outcomes serve as valuable tools for monitoring individuals with SSc in their day-to-day clinical management. The University of California at Los Angeles Scleroderma Clinical Research Consortium GIT 2.0 tool (UCLA GIT 2.0) is a validated questionnaire completed by patients to evaluate the severity of GI symptoms and effect on health-related quality of life (HRQoL) in individuals with SSc. Originally developed in English, it has undergone validation in various languages, with previously identified minimal clinically important differences. This scale has been widely employed as an outcome measure in numerous clinical trials assessing GI treatments for patients with SSc [3, 4].

The Controlling Nutritional Status (CONUT) score helps identify malnutrition and provides valuable information about a patient’s general health and disease prognosis in cardiovascular diseases, cancers, and autoimmune diseases. Increasingly used in rheumatology, the CONUT score has been shown to be related to increasing disease activity and worse patient outcomes [5]. The Prognostic Nutrition Index (PNI) is a clinical device used to assess the nutritional and immune status of a patient. PNI is often applied in various medical conditions, including cancer and chronic diseases, to provide insights into disease severity and prognosis [6].

CONUT and PNI scores have recently been evaluated as indicators of immune-related nutritional status, disease activity and disease prognosis in rheumatic diseases, including antineutrophil cytoplasmic antibody (ANCA)-associated vasculitis, familial Mediterranean fever (FMF) and systemic lupus erythematosus (SLE) [7–9]. We aim to assess whether these evaluation tools, along with the UCLA GIT 2.0 index, can predict GIT involvement in SScpatients, where GI involvement is very common.

## Materials and methods

### Patients and study design

Eighty-two patients admitted to the rheumatology outpatient clinic of our hospital between May 2020 and May 2022 and diagnosed with SSc were included in this cross-sectional study. SSc is diagnosed according to the criteria established by the American College of Rheumatology (ACR) and the European League Against Rheumatism (EULAR) in 2013 [10]. Patients who expressed their willingness to participate in the study were assured that they were fully informed about the evaluation methods and the objectives of the study, and informed consent was obtained. Demographic information, cumulative organ involvement of organs, therapeutic regimens and blood laboratory results, including the acute phase response, were meticulously recorded according to a predefined protocol. Pregnant or breastfeeding patients, patients with known chronic kidney disease or liver failure, patients with known malignancies, patients taking medication for hyperlipidaemia, and patients who had undergone surgery, trauma or haemorrhagic events that could alter inflammatory marker levels in the last 3 months of the study duration were not included in the study.

Ethical committee approved by Marmara University Faculty of Medicine Research Ethics Committee before the study was conducted (approval no: 1429 and date: 03/12/2021). We conducted this study in accordance with the Declaration of Helsinki.

### Clinical measurment data collection

The University of California, Los Angeles Scleroderma Clinical Research Consortium Gastrointestinal Tract (UCLA SCTC GIT) 1.0 questionnaire was first described by Khanna et al. to assess the severity of GI symptoms and their impact on quality of life in patients with SSc. In 2009, this questionnaire was revised and is now known as the UCLA SCTC GIT 2.0 questionnaire [3]. This tool consists of a total of 34 items with subscales evaluating seven categories (reflux, distension, diarrhoea, faecal incontinence, constipation, emotional well-being and social functioning). These subscales, along with the total GIT scores, aim to gauge the quality of life and severity of GIS symptoms in patients [11]. Scores for all subscales except diarrhea and constipation fall within the range of 0.00 to 3.00; meanwhile, the scores for constipation and diarrhea subscales range between 0.00 and 2.00 and 0.00 to 2.50, respectively. Additionally, a “GIS involvement severity score” can be derived using UCLA SCTC GIT 2.0, ranging from 0.00 to 2.83 and calculated using all subscales except constipation. The study specifically evaluated the validity and reliability of the UCLA SCTC GIT 2.0 questionnaire in the Turkish population [4].

Disease activity was evaluated utilizing the criteria established by the European Scleroderma Study Group (EScSG), designed to differentiate the active and inactive disease states. It consists of ten items, each of which is assessed on a 10-point scale to calculate an overall activity index. Initially, the index assigns specific weights to each criterion to reflect activity levels in various systems or organs. Activity scoring and EScSG assessments performed according to the measurement protocol determined in this study. Patients scoring equal to or exceeding the cutoff point of ≥ 2.5 were categorized as having active disease [12].

This modified Rodnan Skin Score (mRSS) involves the assessment of thickness of the skin in 17 regions, including the upper arms, forearms, hands, fingers, chest, abdomen, face, upper legs, lower legs and feet. In each region, the skin is manually palpated by gently squeezing or rolling it between the thumb and index finger. Scoring ranges from 0 to 3, with 0 representing normal thickness, 1 indicating mild thickening, 2 denoting moderate thickening, and 3 signifying severe thickening. The maximum mRSS score is 51, calculated by summing all individual scores [13].

Fatigue was evaluated utilizing the Multidimensional Assessment of Fatigue (MAF) scale, which comprises 16 items designed to measure fatigue across four dimensions [14].

### Nutritional indices and calculation

Basic laboratory parameters used in disease follow-up were employed to calculate PNI and CONUT scores. To calculate PNI, the formula (0.005 x lymphocyte count + 10 x serum albumin [g/dL]) was used [6]. CONUT score obtained from serum albumin, total cholesterol level and peripheral blood lymphocyte count. Each variable in the formula was scored into four groups: albumin (≥ 3.5 = 0, 3.0-3.4 = 2, 2.5–2.9 = 4, < 2.5 = 6), total lymphocyte count (≥ 1600 = 0, 1200–1599 = 1, 800–1199 = 2, < 800 = 3) and serum total cholesterol (≥ 180 = 0, 140–179 = 1, 100–139 = 2, < 100 = 3). The score of CONUT was determined by summing the values of these variables. The total score, indicating the level of malnutrition, was classified as follows: 0–1 for normal, 2–4 for mild, 5–8 for moderate, and 9–12 for severe malnutrition [5]. In our study, we evaluated CONUT scores of 2 and above as indicative of malnutrition.

### Statistical analysis

SPSS version 26.0 statistical software (IBM, Chicago, USA) was used for analysis of the data. The normality of the datasets was assessed with Shapiro-Wilk tests. Categorical variables were expressed as numbers and percentages, quantitative variables showing normal distribution were expressed as mean ± standard deviation (SD), and those not showing normal distribution were expressed as median, minimum and maximum. For normally distributed parameters, independent t tests were used to comparing the two groups. Group comparisons for parameters that did not show normal distribution were performed using Mann-Whitney U tests. Statistical differences in categorical variables were analysed using Fisher exact or chi-square tests. To examine the correlations among the variables, Spearman and Pearson correlation tests were conducted. Statistically significant p value < 0.05 was considered statistically significant.

## Results

**Fig. 1:**
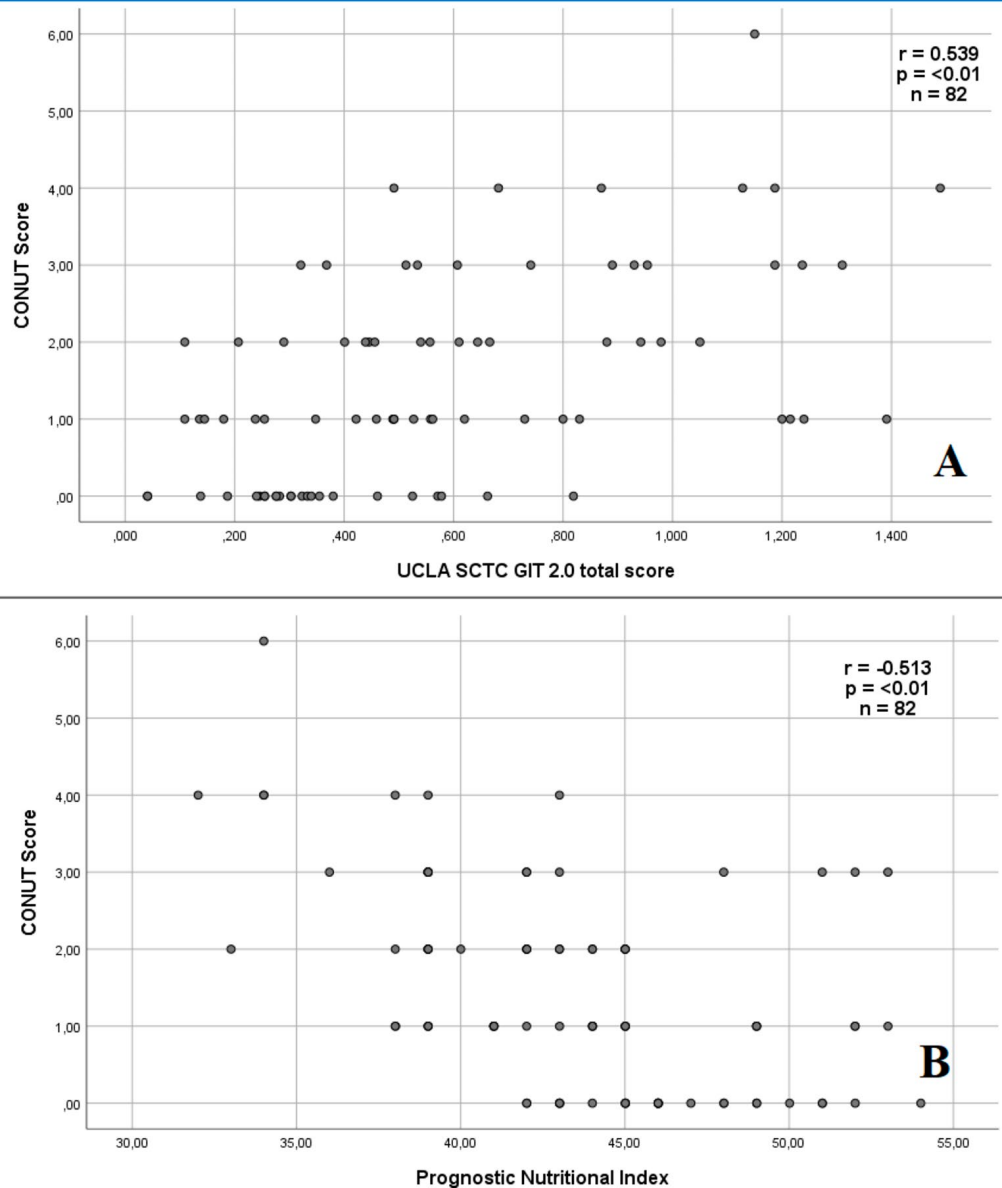
**A** Correlation analysis between CONUT Score and UCLA SCTC GIT 2.0 total score. **B** Correlation analysis between CONUT Score and PNI. CONUT indicates the Controlling Nutritional Status; UCLA SCTC GIT, the University of California, Los Angeles Scleroderma Clinical Trial Consortium Gastrointestinal Tract; PNI indicates Prognostic Nutritional Index

A totally 82 patients with SSc were included in the study, the mean age of the population was 49.38 ± 12.97 years and 80.5% were woman. They were divided into two groups: CONUT score = 0–1 (normal) and CONUT score ≥ 2 (malnutrition). Limited cutaneous SSc was significantly higher in the normal group. EScSG activity indexes were determined as 2.61 ± 1.94 in the entire population and was not significantly different of the groups. Clinical manifestations, outcome measures and treatment status of the patients are given in Table 1 and no significant difference was found except for gastroesophageal reflux disease, VAS handicap, calcium channel blocker and ACE inhibitor use. Laboratory parameters and autoantibodies are given in Table 2 and platelet count, lymphocyte, total cholesterol and albumin were found to be significantly lower in the malnutrition group. CONUT score and PNI were found to be 1.45 ± 1.35 and 43.59 ± 5.01 in the whole population, respectively. UCLA SCTC GIT 2.0 is given in Table 3 and total, reflux, distension, social function and emotional wellbeing were found to be significantly higher in the malnutrition group. The correlation between CONUT score and UCLA SCTC GIT 2.0 subparameteinical parameters is given in Table 4. CONUT score had positive moderate correlation with UCLA SCTC GIT 2.0 total (*r* =.539; *p* <.01) and negative moderate correlation with PNI (*r* = −.513; *p* <.01), respectively (Fig. 1).

**Table 1:** Baseline characteristics, clinical manifestations, outcome measures and treatment status of patients with systemic sclerosis according to the controlling nutritional status score

	All patients *n* = 82	Normal (CONUT = 0–1) *n* = 47	Malnutrition (CONUT ≥ 2) *n* = 35	*p* value
Age (years; mean SD)	49.38 SD 12.97	48.70 SD 11.98	50.29 SD 14.33	0.588
Female, gender, n (%)	66 (80.5%)	35 (74.5%)	31 (88.6%)	0.111
Disease symptoms duration (month; median/min-max)	89 (6–528)	75 (12–406)	96 (6–528)	0.175
EScSG activity indexes (median/min-max)	2.0 (0.5–9.0)	1.5 (0.5–8.5)	2.5 (0.5–9.0)	0.078
**Limited cutaneous SSc, n (%)**	**39 (47.6**%**)**	**27 (57.4**%**)**	**12 (34.3**%**)**	**0.038**
Diffuse cutaneous SSc, n (%)	39 (47.6%)	19 (40.4%)	20 (57.1%)	0.133
The overlap of systemic sclerosis, n (%)	4 (4.9%)	1 (2.1%)	3 (8.6%)	0.308
*Clinical manifestations*				
Raynaud’s phenomenon, n (%)	76 (92.7%)	42 (89.4%)	33 (97.1%)	0.393
Digital ulcers, n (%)	34 (41.5%)	22 (46.8%)	12 (34.3%)	0.255
Sclerodactyly, n (%)	80 (97.6%)	45 (95.7%)	35 (100.0%)	0.505
Telangiectasias, n (%)	78 (95.1%)	45 (95.7%)	33 (94.3%)	0.762
Skin atrophy, n (%)	45 (54.9%)	22 (46.8%)	23 (65.7%)	0.089
Scleredema, n (%)	25 (30.5%)	11 (23.4%)	14 (40.0%)	0.106
Calcinosis cutis, n (%)	18 (22.0%)	8 (17.0%)	10 (28.6%)	0.211
Synovitis, n (%)	30 (36.6%)	13 (27.7%)	17 (48.6%)	0.052
Flexion contractures, n (%)	12 (14.6%)	5 (10.6%)	7 (20.0%)	0.235
Tendon friction rubs, n (%)	7 (8.5%)	4 (8.5%)	3 (8.6%)	0.992
Proximal muscle weakness, n (%)	5 (6.1%)	2 (4.3%)	3 (8.6%)	0.646
**Gastroesophageal reflux disease, n (%)**	**51 (62.2**%**)**	**24 (51.1**%**)**	**27 (77.1**%**)**	**0.016**
Dysfagia, n (%)	46 (56.1%)	23 (48.9%)	23 (65.7%)	0.130
Vomiting, n (%)	10 (12.2%)	3 (6.4%)	7 (8.5%)	0.089
Diarrhea, n (%)	15 (18.3%)	6 (12.8%)	9 (25.7%)	0.134
Constipation, n (%)	27 (32.9%)	13 (27.7%)	14 (40.0%)	0.240
Pulmonary hypertension, n (%)	18 (22.0%)	8 (17.0%)	10 (28.6%)	0.211
Interstitial lung disease, n (%)	33 (40.2%)	15 (31.9%)	18 (51.4%)	0.075
Arrhythmia, n (%)	5 (6.1%)	2 (4.3%)	3 (8.6%)	0.646
Syncope, n (%)	1 (1.2%)	0 (0%)	1 (2.9%)	0.427
Hypertension, n (%)	1 (1.2%)	0 (0%)	1 (2.9%)	0.427
*Outcome measures*				
The Patient’s skin VAS (mean SD)	50.6 SD 22.9	48.1 SD 20.8	54.0 SD 25.5	0.251
The Physician’s skin VAS (median/min-max)	50 (0–100)	50 (0–100)	60 (0–100)	0.680
The Patient’s VAS score for RP (median/min-max)	50 (0–90)	50 (0–90)	50 (0–90)	0.336
The Physician’s VAS score for RP (median/min-max	50 (0–90)	50 (0–90)	50 (0–80)	0.635
VAS disability (median/min-max)	50 (0–100)	50 (0–90)	60 (0–100)	0.088
**VAS handicap (**median/min-max**)**	**50 (0–100)**	**50 (0–90)**	**60 (0–100)**	**0.042**
The Patient’s VAS Pain (median/min-max)	50 (0–100)	50 (0–90)	50 (0–100)	0.078
The Physician’s VAS pain (median/min-max)	50 (0–90)	50 (0–90)	50 (0–80)	0.161
Morning stiffness, minutes (median/min-max)	10 (0–150)	10 (0–120)	15 (0–150)	0.261
Tender joint count (median/min-max)	1.5 (0–10)	1 (0–10)	2 (0–10)	0.945
Swollen joint count (median/min-max)	0 (0–8)	0 (0–3)	0 (0–8)	0.664
Digital ulcers count (median/min-max)	0 (0–5)	0 (0–5)	0 (0–4)	0.800
Pitting scars count (median/min-max)	1 (0–12)	1 (0–12)	2 (0–10)	0.408
Modified Rodnan skin score (median/min-max)	18 (2–51)	18 (2–51)	18 (2–46)	0.778
HAQ-DI (median/min-max)	1.67 (0–47)	1.7 (0–34)	1.65 (0–47)	0.490
MAF scale (mean SD)	30.37 SD 10.99	30.02 SD 10.33	30.85 SD 11.96	0.737
*Treatment*				
**Calcium channel blocker, n (%)**	**67 (81.7**%**)**	**44 (93.6**%**)**	**23 (65.7**%**)**	**0.001**
**ACE inhibitor, n (%)**	**16 (19.5**%**)**	**5 (10.6**%**)**	**11 (31.4**%**)**	**0.019**
Iloprost, n (%)	8 (9.8%)	5 (10.6%)	3 (8.6%)	0.755
Bosentan, n (%)	13 (15.9%)	7 (14.9%)	6 (17.1%)	0.783
PDE-5 inhibitor, n (%)	10 (12.2%)	4 (8.5%)	6 (17.1%)	0.237
Metotrexate, n (%)	30 (36.6%)	19 (40.4%)	11 (31.4%)	0.403
Mycophenolate mofetil, n (%)	35 (42.7%)	19 (40.4%)	16 (45.7%)	0.632
Azathioprine, n (%)	6 (7.3%)	2 (4.3%)	4 (11.4%)	0.394
Rituximab, n (%)	2 (2.4%)	1 (2.1%)	1 (2.9%)	0.832
Cyclophosphamide, n (%)	4 (4.9%)	1 (2.1%)	3 (8.6%)	0.308
Corticosteroid, n (%)	54 (65.9%)	31 (66.0%)	23 (65.7%)	0.982
Hydroxychloroquine, n (%)	40 (48.8%)	21 (44.7%)	19 (54.3%)	0.389

*Abbreviations* CONUT, the Controlling Nutritional Status; SD, standard deviation; min, minimum; max, maximum; EScSG, The European Systemic sclerosis study group; SSc, systemic sclerosis; VAS, Visual Analogue Scale; RP, Raynaud’s phenomenon; HAQ-DI, Health Assessment Questionnaire-Disability Index; MAF, Multidimensional Assessment of Fatigue; ACE, angiotensin-converting enzyme; PDE, phosphodiesterase

**Table 2:** Autoantibodies and laboratory findings of patients with systemic sclerosis according to the controlling nutritional status score

	All patients *n* = 82	Normal (CONUT = 0–1) *n* = 47	Malnutrition (CONUT ≥ 2) *n* = 35	*p* value
*Autoantibodies*				
Anti-Scl 70, n (%)	49 (59.8%)	31 (66.0%)	18 (51.4%)	0.185
Anti-CENP-B antibody, n (%)	23 (28.0%)	11 (23.4%)	12 (34.3%)	0.278
Anti-Ro, n (%)	3 (3.7%)	1 (2.1%)	2 (5.7%)	0.573
Anti-Sm-RNP, n (%)	52(6.1%)	0 (0%)	2 (5.7%)	0.179
Anti-Jo1, n (%)	1 (1.2%)	0 (0%)	1 (2.9%)	0.427
Anti-Ro-52, n (%)	7 (8.5%)	3 (6.4%)	4 (11.4%)	0.453
*Laboratory findings*				
White blood cell count, cells/µL (median/min-max)	7.1 (4.0–14.8)	7.4 (4.0–12.9)	6.1 (4.2–14.8)	0.081
**Lymphocyte, cells/µL (median/min-max)**	**1.6 (0.8–3.0)**	**1.8 (1.2–3.0)**	**1.2 (0.8–2.3)**	**< 0.001**
Hemoglobin, g/dL (mean SD)	11.9 SD 1.6	12.0 SD 1.7	11.7 SD 1.5	0.344
**Platelet count, cells/µL (median/min-max)**	**255 (118–707)**	**284 (118–707)**	**228 (144–465)**	**0.007**
ESR, mm/h (median/min-max)	23 (2–120)	28 (4–72)	23 (2–120)	0.725
CRP, mg/L (median/min-max)	3.17 (0.18–59.0)	3.38 (0.65–59.0)	2.96 (0.18–52.1)	0.414
**Total cholesterol, mg/dL (median/min-max)**	**178 (115–298)**	**198.8 SD 34.5**	**156.6 SD 36.1**	**< 0.001**
**Albumin, gr/L (mean SD)**	**43.58 SD 5.01**	**45.40 SD 4.18**	**41.14 SD 5.05**	**< 0.001**

*Abbreviations* CONUT, the Controlling Nutritional Status; Anti-Scl 70, Antibodies against Scl-70 antigen; Anti-CENP-B, Antibodies against centromere protein B; Anti-Ro, Antibodies against Ro antigens; Anti-Sm-RNP, Antibodies against Smith antigen and ribonucleoprotein; Anti-Jo1, Anti-Histidyl-tRNA Synthetase; Anti-Ro-52, Antibodies against Ro-52; min, minimum; max, maximum; SD, standard deviation; ESR, erythrocyte sedimentation rate; CRP, C-reactive protein

**Table 3:** Malnutrition and UCLA SCTC GIT 2.0 scores patients with systemic sclerosis according to the controlling nutritional status score

	All patients *n* = 82	Normal (CONUT = 0–1) *n* = 47	Malnutrition (CONUT ≥ 2) *n* = 35	*p* value
**CONUT score (median/min-max)**	**1 (0–6)**	**0 (0–1)**	**3 (2–6)**	**< 0.001**
**Prognostic Nutritional Index (mean SD)**	**43.59 SD 5.01**	**45.41 SD 4.18**	**41.14 SD 5.05**	< 0.001*
UCLA SCTC GIT 2.0				
**Total (median/min-max)**	**0.50 (0.04–1.49)**	**0.36 (0.04–1.39)**	**0.67 (0.11–1.49)**	**< 0.001**
**Reflux (median/min-max)**	**0.75 (0–2.75)**	**0.63 (0–1.63)**	**0.88 (0–2.75)**	**< 0.001**
**Distension (median/min-max)**	**1 (0 -2.5)**	**0.75 (0 -1.5)**	**1.25 (0.25–2.5)**	**< 0.001**
Soilage (median/min-max)	0 (0–2)	0 (0–2)	0 (0–2)	0.305
Diarrhoea (median/min-max)	0 (0–1.5)	0 (0–1.5)	0 (0–1.5)	0.286
Constipation (median/min-max)	0.29 (0–1.75)	0.5 (0–1.5)	0.25 (0–1.75)	0.581
**Social function (median/min-max)**	**0.64 (0–1.64)**	**0.32 (0–1.47)**	**0.80 (0–1.64)**	**0.002**
**Emotional wellbeing (median/min-max)**	**0.33 (0–1.43)**	**0.11 (0–1.10)**	**0.44 (0–1.43)**	**0.008**

*Abbreviations* UCLA SCTC GIT, the University of California, Los Angeles Scleroderma Clinical Trial Consortium Gastrointestinal Tract; CONUT, the Controlling Nutritional Status; min, minimum; max, maximum; SD, standard deviation

**Table 4:** Correlation of UCLA SCTC GIT 2.0 scores, clinical variables and laboratory findings with the controlling nutritional status score

	CONUT score *r*/rho	
UCLA SCTC GIT 2.0		
Total	0.530**	rho
Reflux	0.543**	rho
Distension	0.556**	rho
Soilage	0.218*	rho
Diarrhoea	0.209	rho
Constipation	0.003	rho
Social function	0.460**	rho
Emotional wellbeing	0.452**	rho
Age (years)	0.129	r
EScSG activity indexes	0.263*	rho
Hemoglobin, g/dL	-0.227*	r
Platelet count, cells/µL	-0.215	rho
ESR, mm/h	0.088	rho
CRP, mg/L	-0.042	rho
Prognostic Nutritional Index	-0.513**	r
Modified Rodnan skin score	0.087	rho
HAQ-DI	0.133	rho
MAF scale	0.075	r

*Abbreviations* UCLA SCTC GIT, the University of California, Los Angeles Scleroderma Clinical Trial Consortium Gastrointestinal Tract; CONUT, the Controlling Nutritional Status; EScSG, The European Systemic sclerosis study group; ESR, erythrocyte sedimentation rate; CRP, C-reactive protein; HAQ-DI, Health Assessment Questionnaire-Disability Index; MAF, Multidimensional Assessment of Fatigue

*P <.05

**P <.01

r Pearson corelation / rho Spearman’s corelation

## Discussion

In this study, evaluating the feasibility of using CONUT and PNI malnutrition indices as indicators of GI involvement in patients with SSc was aimed. These indices may serve as useful tools to identify patients at risk of potentially severe GI symptoms. The integration of these easily calculated indices based on routine laboratory parameters into clinical practice will provide potential advantages for SSc management, particularly in identifying patients at higher risk of GI involvement. Patients identified as being at nutritional risk should be evaluated with upper GI endoscopy, manometry, or other diagnostic evaluations to elucidate GI pathology. We demonstrated the correlation between these malnutrition scores, which can be calculated using simple laboratory parameters routinely collected during patient follow-up, and the UCLA GIT score, an outcome measure validated by numerous studies in this field. Our data showed that using the UCLA GIT 2.0 scale, along with the malnutrition indices CONUT and PNI, can help clinicians identify patients who may need further investigation.

SSc can affect any part of the GI system, with oesophageal and intestinal symptoms occurring in 90% and 40–70% of patients, respectively. GI problems significantly impact quality of life, leading to malnutrition, malabsorption, and electrolyte imbalances [15]. While invasive techniques like endoscopy and manometry are gold standards for diagnosing GI involvement [16, 17], their routine use is often limited by logistical and procedural challenges. The UCLA GIT 2.0 tool offers a reliable, non-invasive method for assessing GI symptoms, highlighting the need for simple and accessible screening approaches in SSc patients [3, 4].

In SSc, malnutrition may be caused by a number of factors related to the disease itself and its complications. Especially in GI involvement, dysphagia due to oesophageal dysmotility, early feeling of satiety, nausea and decreased food intake due to gastroparesis are important causes of malnutrition [2]. Limited mounth opening secondary to skin involvement can make it difficult to eat. Chronic systemic inflammation may also have detrimental effects on nutrition and lead to muscle wasting and weight loss. Patients often have comorbid depression and anxiety, which can reduce appetite and motivation to eat. In addition, medications such as immunosuppressants and corticosteroids used to manage SSc can have side effects such as nausea, vomiting and decreased appetite [18]. Different studies have reported that the prevalence of malnutrition, which is an independent risk factor for mortality in SSc, is between 8% and 55% [19–21]. In our study, malnutrition was detected 32 out of 82 patients according to the CONUT score. In a study analyzing the causes of death in SSc patients registered in the European Scleroderma Trials and Research (EUSTAR) database, the overall frequency of deaths due to GI involvement was 3.5% (*n* = 1072/11193, 9.6%). The difference between disease subgroups showed that patients with limited cutaneous disease (2%) had limited cutaneous disease less frequently than patients with diffuse cutaneous disease (6%), but no significant difference was found [22]. Malnutrition (34.3%) was significantly lower in patients with limited skin involvement in this study (*p* =.038). In a study assessing the prevalence of symptoms related to gastrointestinal involvement and malnutrition in systemic sclerosis (SSc), a significant positive correlation was identified between disease duration and the occurrence of malnutrition, indicating that malnutrition becomes more prevalent as the disease progresses [23]. In our study, disease symptom duration (months) was detected to be longer in patients with CONUT score ≥ 2, but there was no statistical significance. In this study, gastroesophageal reflux disease *n* = 51 (62.2%) and dysphagia *n* = 46 (56.1%) were the dominant GI complaints. Gastroesophageal reflux disease was significantly more common in patients with malnutrition. On the other hand, there were no differences in clinical symptoms, autoantibody subtypes, immunosuppressive use or disease activity and severity between the two groups.

In SSc, for malnutrition assessment, there is no defined screening or clinical pathway. In many studies, anthropometric measurements such as Body Mass Index (BMI), the Global Leadership Initiative on Malnutrition (GLIM) criteria, and the European Society of Clinical Nutrition and Metabolism (ESPEN) guidelines have been used with different indices to screen for malnutrition [24]. In our study, we used the CONUT score as a screening tool for malnutrition. The CONUT score can be calculated through simple laboratory evaluations and provides a quick and easy assessment of the nutritional status of patients in clinical settings. In a study evaluating the usability of the CONUT score to predict poor outcomes of ANCA-associated vasculitis, they reported that the score is an indicator of all-cause mortality in vasculitis patients [7]. In the study investigating the relationship between CONUT and PNI in subclinical inflammation in FMF patients and their association with the long-term prognosis of the disease, FMF patients with complications of amyloidosis were found to have high CONUT scores and low PNI [8]. This finding reflects the observed inverse correlation between CONUT and PNI scores in our study, which is consistent with their distinct clinical interpretations. Higher CONUT scores indicate worse nutritional status, while higher PNI scores reflect better nutritional and immune status. These indices, therefore, complement each other in providing a holistic view of malnutrition by capturing both deficiencies and preserved nutritional and immune components.

In a recent study examining the effect of malnutrition on mortality and prognosis in patients with Sjögren’s syndrome-associated interstitial lung disease (SS-ILD), it was found that mortality rates were significantly higher in patients with low PNI scores. it was also shown that the use of simple nutritional indicators such as PNI in clinical practice may be useful in evaluating the prognosis of these patients [25]. The study analyzed the relationship between the PNI, CONUT score, the nutritional risk index (NRI), clinical disease activity and damage in 173 patients with SLE. Authors concluded by using serum albumin and lymphocyte count, PNI and NRIhave the potential to be beneficial in clinical practice as simple, low-cost markers for follow-up of disease activity in patients with SLE. However, the CONUT score was not found to be significant by logistic regression analysis [9]. On the contrary, five different nutritional indices, including CONUT score, PNI, NRI, neutrophil-to-lymphocyte ratio and BMI, were assessed in relation to disease activity and ESRD in 207 patients with renal biopsy-proven lupus nephritis. Among these indices, PNI and CONUT score were found to correlate better with disease activity [26].

It also demonstrated that the PNI reliably reflects disease activity in rheumatoid arthritis (RA) patients, further supporting its use as a practical and accessible tool in clinical settings [27].These findings align with our study, where moderate correlations between CONUT and PNI scores and the UCLA GIT 2.0 index were observed in systemic sclerosis (SSc) patients. In a study evaluating Bioelectrical Impedance Vector Analysis (BIVA) to assess nutritional status in patients with systemic sclerosis, it was emphasised that hypoalbuminemia and upper gastrointestinal symptoms such as reflux and early satiety were associated with significant changes in BIVA parameters. While our study employed different assessment methods, these findings are consistent with our observations, supporting the notion that malnutrition is strongly linked to GI tract involvement in systemic sclerosis [28]. All these results not only reinforce the interconnectedness of malnutrition and systemic inflammation in autoimmune diseases, but also emphasise the potential of these indices to aid in the assessment of disease activity, outcomes related to disease involvement and mortality risk.

Our study has some limitations. Long term results are lacking because of cross-sectional nature of study. Recognising these limitations, the findings of the study should be interpreted with caution and future research can be directed to address these gaps. Results show that higher CONUT and lower PNI scores are associated with higher GI symptom severity, but due to the cross-sectional nature of our study, it cannot confirm whether malnutrition contributes to the progression of GI complications or occurs as a consequence of existing GI involvement. This is a limitation of our study and a longitudinal study design will be crucial to further validate the usefulness of these indices as prognostic tools in clinical practice.

There is a need for future studies using larger, more diverse populations and including objective measures of GI involvement.

Our findings suggest that these malnutrition indices could be useful in daily clinical practice. By incorporating these laboratory parameters alongside the UCLA GIT score, healthcare providers can better assess GI involvement and thus be useful in identifying patients for invasive testing.

## Electronic supplementary material

Below is the link to the electronic supplementary material.

Supplementary Material 1

## Data Availability

Data supporting the findings of this study are available from the corresponding author upon reasonable request. Data sharing complies with institutional and ethical guidelines.

## Declarations

### Note on related congress abstracts and publication

This study has not been previously presented at any congress or conference.
